# The steady-state visual evoked potential (SSVEP) reflects the activation of cortical object representations: evidence from semantic stimulus repetition

**DOI:** 10.1007/s00221-020-05992-8

**Published:** 2020-12-14

**Authors:** Elise L. Radtke, Ulla Martens, Thomas Gruber

**Affiliations:** 1 Institute of Psychology, Osnabrück University, Seminarstraße 20, 49074 Osnabrück, Germany; 2 DRK Norddeutsches Epilepsiezentrum für Kinder und Jugendliche, Henry-Dunant-Straße 6-10, 24223 Schwentinental, Germany

**Keywords:** Object representations, Repetition suppression, Semantic priming, SSVEP

## Abstract

We applied high-density EEG to examine steady-state visual evoked potentials (SSVEPs) during a perceptual/semantic stimulus repetition design. SSVEPs are evoked oscillatory cortical responses at the same frequency as visual stimuli flickered at this frequency. In repetition designs, stimuli are presented twice with the repetition being task irrelevant. The cortical processing of the second stimulus is commonly characterized by decreased neuronal activity (repetition suppression). The behavioral consequences of stimulus repetition were examined in a companion reaction time pre-study using the same experimental design as the EEG study. During the first presentation of a stimulus, we confronted participants with drawings of familiar object images or object words, respectively. The second stimulus was either a repetition of the same object image (perceptual repetition; PR) or an image depicting the word presented during the first presentation (semantic repetition; SR)—all flickered at 15 Hz to elicit SSVEPs. The behavioral study revealed priming effects in both experimental conditions (PR and SR). In the EEG, PR was associated with repetition suppression of SSVEP amplitudes at left occipital and repetition enhancement at left temporal electrodes. In contrast, SR was associated with SSVEP suppression at left occipital and central electrodes originating in bilateral postcentral and occipital gyri, right middle frontal and right temporal gyrus. The conclusion of the presented study is twofold. First, SSVEP amplitudes do not only index perceptual aspects of incoming sensory information but also semantic aspects of cortical object representation. Second, our electrophysiological findings can be interpreted as neuronal underpinnings of perceptual and semantic priming.

## Introduction

Bottom-up processing of visual object information goes from simple feature representations (Tanaka 1993) to activating object representation networks and subsequent object recognition. In contrast to object perception, as for example, in unfamiliar objects, object recognition is achieved if cortical networks are activated and the semantic aspects can be accessed (Martens, Wahl et al. 2012a, b; Patterson et al. 2007, i.e., “semantic memory” or “conceptual knowledge” can be accessed). To investigate this activation of semantic associations, we used the steady-state visual evoked potentials (SSVEPs), that is, an oscillatory cortical response at the same frequency as a visual stimulus flickered at this frequency (Regan 1989) in combination with a stimulus repetition design. The goal of this research was twofold. First, we intended to provide evidence that SSVEPs reflect the activation of cortical object representations. To that end, we examined SSVEPs during a semantic stimulus repetition task (SR) versus a perceptual stimulus repetition task (PR). Second, we aimed at extracting activation patterns that are exclusive to SR and, therefore, reflect the activation of semantic aspects of cortical object representations.

The genesis of SSVEPs is not yet explained sufficiently. One theory suggests that SSVEPs can be explained by a linear superposition of transient event-related responses originating in early sensory areas (Capilla et al. 2011). One first indication against this theory is that although the primary visual cortex (V1) is the strongest source of SSVEPs, it is not exclusively responsible for the SSVEP generation but additional dipoles are involved (Vialatte et al. 2010). An alternative theory proposes that SSVEP amplitudes reflect higher-level cognitive processes. The findings that stimulus flicker can entrain activity beyond the stimulated sensory area (Srinivasan et al. 2007), that the stimulus flicker frequency can modulate performance in cognitive tasks (Thut et al. 2011; Williams et al. 2006), that the brain retains a memory trace of the flicker frequency of tagged items (Wimber et al. 2012), and that the entrained oscillations continue to persist for a few circles after the end of stimulation (Halbleib et al. 2012; Ross et al. 2005) speak for the involvement of broader networks that operate via endogenous responses (for a review see e.g., Thut et al. 2011; Zoefel et al. 2018). Furthermore, in a familiar/unfamiliar object recognition task it was shown, that SSVEPs are sensitive to stimuli’s semantic content (Kaspar et al. 2010) and can be used to study the mechanism underlying scene perception (Martens et al. 2011). The present study aims to shed further light on the role of SSVEPs in processing semantic aspects of objects by using a stimulus repetition design.

In stimulus repetition designs, stimuli are presented twice with a randomized number of intervening stimuli. Behaviorally, repetition of an identical or similar stimulus is associated with improved accuracy or reaction times, known as priming effects (Schacter and Buckner 1998; Tulving and Schacter 1990). Typically, these repetition priming effects are accompanied by a reduction of neural firing, decreased BOLD responses, and a decrease in specific EEG markers in response to the repeated stimulus (repetition suppression; Friese et al. 2012; Grill-Spector et al. 2006; Lebreton et al. 2012; Weigelt et al. 2008). Repetition suppression is thought to reflect a “sharpening” of cortical networks representing a stimulus (Gotts et al. 2012; Wiggs and Martin 1998). Sharpening in turn is associated with the behavioral priming effect based on more efficient stimulus representations. Importantly, a repetition suppression effect can also be observed for the SSVEP when repeating pictures of familiar objects (Martens and Gruber 2012). In the present study, we used object words as prime that are always repeated as object images. This rationale for this approach is explained in the following.

As mentioned before, the first goal of the present study was to provide further evidence that SSVEPs reflect higher-level cortical processing. To that end, we repeated a word by its semantically related picture (SR). If SSVEPs were simply an overlap of cortical responses in early visual areas, SR should not result in repetition suppression because both stimuli share no sensory similarities. In contrast, if the two stimuli share certain aspects within their cortical representation, repetition suppression should occur during SR. In other words, if the SSVEP amplitude reflects activity within downstream cortical areas, one has to expect a repetition suppression effect during SR due to the overlapping semantic aspects of the cortical stimulus representations of a word and the representation of its associated picture.

The second goal of the present study was to investigate the morphology of the semantic aspects of a cortical object representation as indexed by SSVEP repetition effects. To this end, additionally to SR we used PR by presenting a picture twice. We expected different topographies, in terms of repetition suppression in PR versus SR. Furthermore, we estimated the cortical sources of SR- versus PR-related suppression effects to elaborate on the SSVEP adaptations which are exclusive to SR. In a companion behavioral study, we controlled for the occurrence of behavioral priming effects in an experimental design using flickering stimuli.

## Methods

### Participants

Nineteen students from Osnabrück University gave their informed consent and participated in the study. Nine of them were males. Their average age was 23.2 years (SD = 2.3 years). They all had normal or corrected to normal vision and no psychological or neurological disorder, specifically they had no migraine or epilepsy and took no medication. The study was approved by the Ethics Committee of Osnabrück University. One data set was excluded due to excessive artifacts in the EEG—therefore, we analyzed the data of 18 participants.

We conducted a behavioral companion study to assess priming effects during the presentation of our flickering stimuli. For this behavioral pre-study, we tested 18 participants (different participants as in the EEG experiment were used). One participant was excluded due to migraine after the experiment. All of them were females. Their average age was 21.7 years (SD = 7.5 years).

### Stimuli and design

**Fig. 1:**
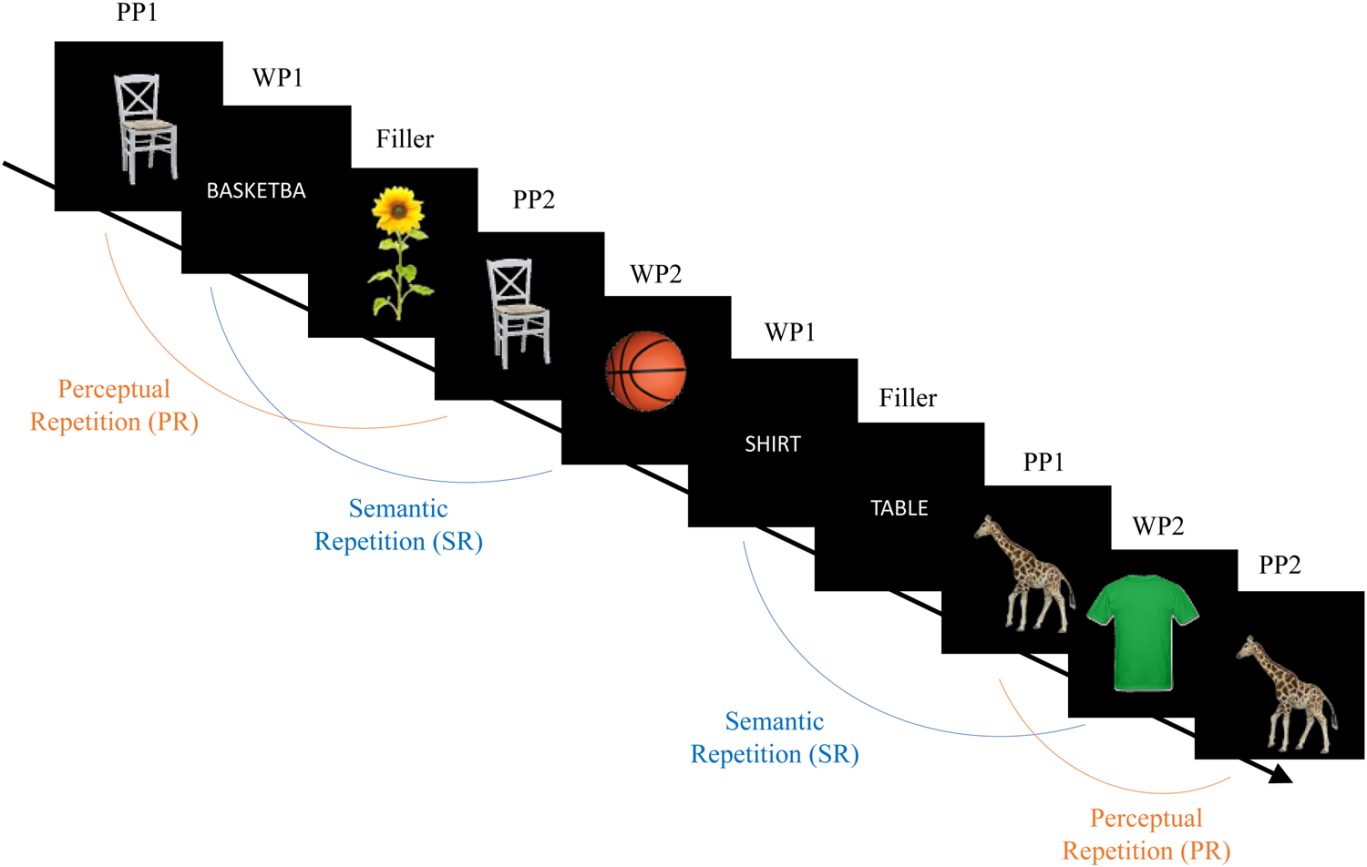
Excerpt of the stimulus sequence with examples of the four experimental conditions (PP1, PP2, WP1, WP2) and a visualization of difference calculations between conditions, that is, semantic repetition (SR) and perceptual repetition (PR). The responses to Fillers and WP1 items were not further analyzed

The experimental design was similar as in the publication by Friese et al. (2012). However, we used a different set of pictures (color instead of black and white) and slightly reduced the total number of trials. In particular, we selected 260 pictures of real-world objects from a comercially available picture library (DVD Hemera Photo Objects 1997). Half of these pictures depicted animate objects (e.g., a giraffe) and the other half showed inanimate objects (e.g., a brush). Of these 260 stimuli, 100 pictures and their corresponding 100 words (max. 15 letters) were chosen randomly for the presentation during the experiment. In our experiment, 100 times, a picture was presented twice (picture repetition; PR) and 100 times the word was repeated by its semantically related picture (semantic repetition; SR). The prime and probe never appeared directly after another, but with one or two intervening stimuli. To reduce expectancy effects concerning the probe presentations, we used 40 words and 40 unrelated pictures that merely served as filler items and had no other function. All words and pictures were presented as a continuous stream of stimuli (see Fig. 1 for an excerpt of the stimulus sequence). This design resulted in six experimental conditions (480 trials): (1) First presentation of a picture (PP1; 100 trials), (2) Second presentation of this picture (PP2; 100 trials), (3) Presentation of a word (WP1; 100 trials), (4) Presentation of the picture corresponding to this word (WP2; 100 trials), (5) Filler_Word_ (40 trials), and (6) Filler_Picture_ (40 trials). Note that only the conditions PP1, PP2, WP2 were used for subsequent analyses. See Table 1 for an overview. Specifically, the difference of the SSVEP elicited by PP1 trials and PP2 trials represents the effects of PR. The difference between PP1 and WP2 trials represents the effects of SR.

**Table 1:** Experimental conditions and differences of interest

Prime	Probe	Analyzed: SSVEP/behavioral reaction after	Condition name	Difference calculations
Picture	Picture	Prime = picture	PP1	
		Probe = picture	PP2	Perceptual repetition (PR) = PP1 − PP2
Word	Picture	Prime = word	WP1 (not further analyzed)	
		Probe = picture	WP2	Semantic repetition (SR) = PP1 − WP2

Each trial (word or picture) consisted of a jittered 500–900-ms baseline period during which a fixation cross was presented, followed by the stimulus for 3000 ms. After the stimulus, a signal cross was presented for 800 ms for the participants to blink if necessary. Participants were asked to avoid eye movements and blinking during the display of the fixation cross and the stimuli. To allow for breaks, the experiment was subdivided into four blocks, each consisting of 120 trials. Prior to the experiment, participants performed a practice block of 80 trials.

Every stimulus was located centrally on a black background and covered a visual angle of 7° × 7°, and the fixation cross covered an angle of 3 × 3°, and stimulus onset was synchronized to the vertical retrace of the monitor. We used a monitor with a refresh rate of 60 Hz. The drawings were presented every 4th frame, that is, at 15 Hz, with a duty cycle 1:3, to elicit a 15 Hz SSVEP. This frequency was selected based on a previous study regarding the relation of SSVEPs to object recognition (Kaspar et al. 2010). To ensure precise timing, we used Matlab and the Psychophysics Toolbox extensions (Brainard 1997).

To maintain the participants’ attention to the stimuli, they had to press the space bar as soon as they detected a magenta dot which was superimposed on some of the words and pictures. 15% of the trials had a magenta dot which appeared at a random position for 120 ms in a time window from 250 to 2750 ms after the object onset. Trials with a magenta dot were excluded from the EEG analyses.

### Behavioral companion study

For the behavioral companion study, we used the same procedure and stimulus material except for the participants’ task. Instead of the dot detection task, they had to decide whether the object is animate or inanimate. We opted for the approach to examine behavioral data in a companion study because in SSVEP studies, it is important that the participants attend to the stimulus all the time. However, in an animate/inanimate judgment task, it is likely that participants withdraw their attention from the stimulus as soon as the animate/inanimate classification results in a decision. Since the animate/inanimate judgement was solely used as an incidental task to detect repetition priming effects, the subjective participants’ classifications as living or non-living were not further analyzed. Furthermore, reaction times below 200 ms and above 2000 ms were considered as outliers and were not included into the statistical analysis.

### Electrophysiological recordings and preprocessing

EEG was recorded using 128 electrodes and a BioSemi Active Two amplification system with a sampling rate of 512 Hz. As reference and ground electrodes, two additional electrodes were used (CMS and DRL; for more info see (https://www.biosemi.com/faq/cms&drl.htm). Eye movements and blinks were measured by vertical and horizontal electro-oculogram. For preprocessing and EEG analysis, we used Matlab 2018a and the EEGLab toolbox version 14 (Delorme and Makeig 2004). In line with several previous SSVEP studies (e.g., Kaspar et al. 2010; Martens and Gruber 2012), the data were segmented into epochs from − 500 to + 3000 ms relative to stimulus onset (baseline was from − 400 to − 200 ms) and artifact corrected by means of the technique known as ‘statistical correction of artifacts in dense array studies’ (SCADS; Junghöfer et al. 2000). Single epochs with excessive eye movements and blinks or > 20 channels containing artifacts were removed. Additionally, some rare artifacts that were not detected by SCADS were eliminated after visual inspection. The average rejection rate of EEG data after artifact correction was approximately 20% of the epochs. Note, that the rejection rates did not significantly differ between conditions. Finally, the data were re-referenced to the average of all electrodes.

### Data analysis

#### Behavioral data

In the companion behavioral priming experiment, we analyzed reaction times of the animate/inanimate judgment by means of a one-way ANOVAs with the factor Condition (PP1 vs. PP2 vs. WP1 vs. WP2) followed by post hoc *t-*tests. In the SSVEP study, we only tracked the percentage of correct responses in the magenta dot detection task.

#### SSVEPs in electrode space

**Fig. 2:**
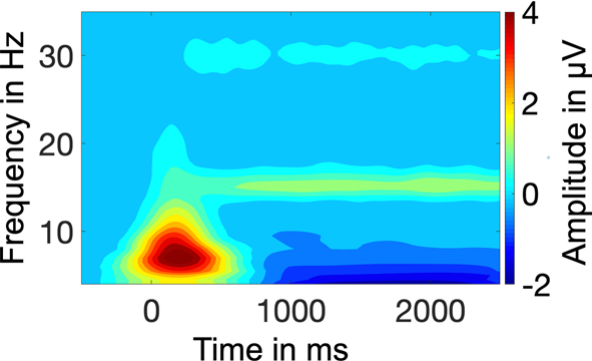
Baseline-corrected time by frequency plot averaged across PP1, PP2, WP2 conditions and all electrodes. An ongoing SSVEP response at 15 Hz is clearly visible

To receive the temporally changing magnitude of the SSVEP at 15 Hz, the EEG signal was spectrally decomposed by means of Morlet wavelet analysis (Bertrand and Pantev 1994) for a family of wavelets ranging from 1 to 30 Hz (approximately 12 cycles per wavelet). The resulting time by frequency representation of the average across all electrodes (Fig. 2) shows that we succeeded in eliciting a 15 Hz response, i.e. the flicker frequency of the external pacemaker. The SSVEP reached a plateau at around 500 ms after stimulus onset and was stable until the end of the epoch. Based on this time by frequency plot, we selected a time window from 500 to 2000 ms after stimulus onset for further analysis.

**Fig. 3:**
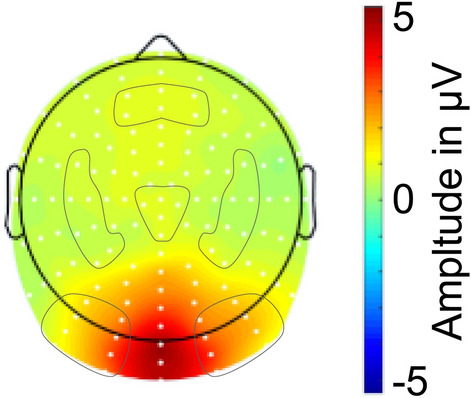
Baseline-corrected topographical distribution of the 15-Hz SSVEP response in the time window from 500 to 2000 ms averaged across PP1, PP2, WP2 conditions

Figure 3 depicts the topographical amplitude distributions within the selected time window averaged across all conditions. The topography is characterized by the SSVEP’s typical maximum over occipital regions. For further statistical analysis, we divided the electrodes into 6 regions as indicated in Fig. 3 and calculated the mean amplitude for electrodes in each region. Prior to the study, we decided to evaluate difference topographies (PP1 minus PP2 representing PR effects and PP1 minus WP2 representing SR effects) to determine appropriate regions for statistical analyses. This is a valid approach as long as the topography of both conditions is taken into account equally, so that it is used as an unbiased approach with respect to differences between the conditions (Keil et al. 2014). Additionally, this approach is justified by the fact that previous SSVEP studies have indeed shown effects at electrode sites that did not correspond to the electrode sites of the maximum SSVEP amplitude in the averaged topography (e.g., Kaspar et al. 2010; Martens et al. 2011; Martens and Hübner 2013; Silberstein et al. 2001).

We conducted a 6 (Regional Mean: frontal, left temporal, right temporal, central, left occipital, right occipital) × 3 (Condition: PP1, PP2, WP2) ANOVA with the averaged amplitude as the dependent variable, post hoc one-way ANOVAs, and post hoc *t-*tests. Note that only SSVEP responses to pictures (PP1, PP2, and WP2) were included in the analysis. WP1 (i.e., words) was not included due to the mere sensory differences of the stimuli in this condition. All ANOVA-related *p* values are Greenhouse–Geisser corrected, because in some analyses, the assumption of sphericity was violated.

### SSVEPs in source space

The SSVEP analysis in source space followed a procedure we successfully used in a previous SSVEP study (Radtke et al. 2020). To localize activation differences in PR versus SR, we used variable resolution electromagnetic tomography (VARETA; Bosch-Bayard et al. 2001). This procedure provides the spatially smoothest intracranial distribution of current densities in source space, which is most compatible with the amplitude distribution in electrode space (Gruber et al. 2006). The inverse solution consisted of 3244 grid points (“voxels”) of a 3D grid (7-mm grid spacing). This grid and the arrangement of 128 electrodes were placed in registration with the average probabilistic MRI brain atlas (“average brain”) produced by the Montreal Neurological Institute (MNI; Evans et al. 1993). To localize the activation difference between two conditions, paired *t-*tests with a significance level of *p* < 0.05 were performed. Activation threshold corrections accounting for spatial dependencies between voxels were calculated by means of random field theory (RFT; Kilner et al. 2005; Worsley et al. 1996). The thresholds for all statistical parametric maps were set to a significance level of *p* < 0.05. Finally, the significant voxels were projected to the cortical surface based on the MNI average brain. Area names for significant voxels were identified by the xjview toolbox (http://www.alivelearn.net/xjview) which uses the automated anatomical labeling toolbox (AAR2; Tzourio-Mazoyer et al. 2002). We conducted two source analyses. (MNI; Evans et al. 1993). One for the PP1–PP2 difference, representing PR effects, and one for the PP1–WP2 difference, representing SR effects.

### Power analyses

To our knowledge, no valid published method exists for power calculations with respect to multi-factor within-ANOVAs. Therefore, we did not calculate an a priori power analysis. Based on the previous SSVEP studies that tested approximately 20 participants, we decided to follow this suggestion to obtain robust results (e.g., Friese et al. 2012; Martens and Gruber 2012). Nonetheless, based on a reviewer’s suggestion, we performed a power analyses with respect to our one-way repeated-measures ANOVAs and the post hoc *t*-tests. Particularly, we conducted a sensitivity analysis with G*Power (Faul et al. 2007). Sensitivity analyses detect the minimum effect size that could reliably yield a statistically significant result, given a specific power. Here, a power of 0.8, an *α* error probability of 0.05, and *n* = 18 participants were entered.

## Results

### Behavioral results

In the companion reaction time study, we obtained the expected priming effects. In particular, all reaction times differed significantly depending on the condition, *F*(1.73, 27.74) = 40.20, *p* < .001. Post hoc *t-*tests revealed that participants responded faster to the animate/inanimate judgment in the PP2 condition (*M* = 781 ms, SD = 16 ms) than in the WP2 condition (*M* = 815 ms, SD = 18 ms), *t*(16) = 5.4, *p* < .001, *d* = 1.3, and in turn faster than in the PP1 condition (*M* = 849 ms, SD = 21 ms), *t*(16) = 4.7, *p* < .001, *d* = 1.14, and in turn faster than in the WP1 condition (*M* = 972 ms, SD = 23 ms), *t*(16) = 8.7, *p* < .001, *d* = 2.11. The faster responses in the WP2 compared to the PP1 condition shows that semantic priming was successful.

In the EEG study, participants showed an averaged detection rate of 90% and error rates did not differ between conditions, *F*(1.79, 28.64) = 1.76, *p* = .193.

### SSVEPs in electrode space

**Fig. 4:**
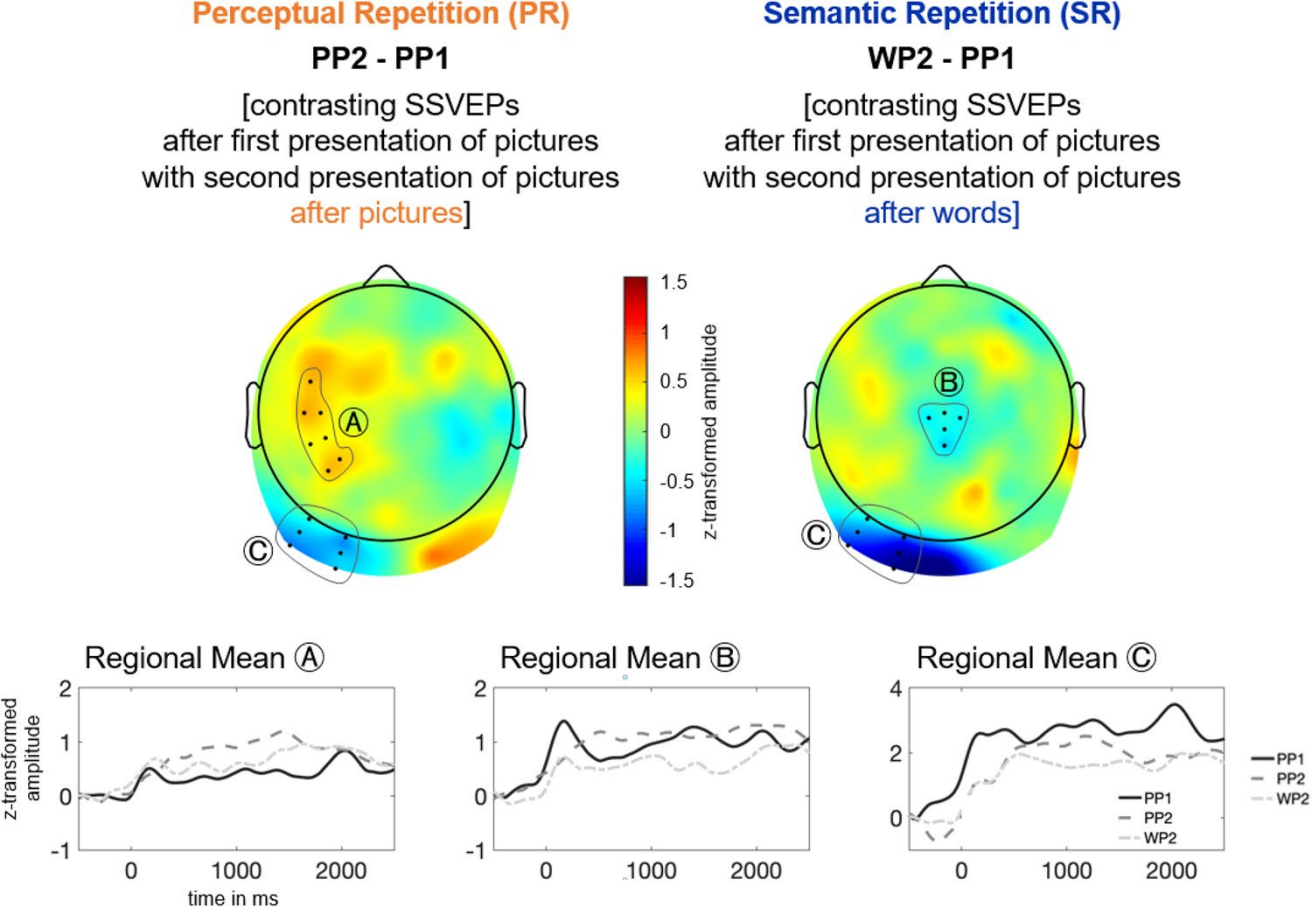
Visualization of results for PR (left) and SR (right). Top: Topographic distribution of activation differences averaged in the time window from 500–2000 ms. Bottom: time course of average amplitudes at selected regional means for the three conditions

Figure 3 shows that the 15-Hz SSVEP amplitude was characterized by a maximum at occipital electrodes and some spreading activation to more anterior sensors. Looking at the different experimental conditions, depicted in Fig. 4, one can see that PR was associated with repetition suppression at the left occipital and repetition enhancement at the left temporal regional mean. SR was associated with repetition suppression at the central and left occipital regional mean. These results of visual inspection were substantiated by statistical analyses.

In the ANOVA, the main effect Condition did not reach significance, *F*(1.78, 30.32) = 1.80, *p* = .185. The main effect Regional Mean, *F*(2.34, 39.85) = 13.48, *p* < .001, $\eta_{p}^{2}$ = .44, was further characterized by a significant interaction effect of Condition x Regional Mean, *F*(2.34, 39.85) = 13.48, *p* = .002, $\eta_{p}^{2}$ = .18. To break down this interaction effect, we computed six one-way ANOVAs, that is, one for each Regional Mean. At the frontal, *F*(1.61, 27.33) = 0.03, *p* = .948, left temporal, *F*(1.43, 24.28) = 3.66, *p* = .054, $\eta_{p}^{2}$= .18, right temporal, *F*(1.83, 31.10) = 0.91, *p* = .404, and right occipital, *F*(1.80, 30.68) = 1.92, *p* = .167, areas, the condition effect did not reach significance. The SSVEP amplitudes differed sginficantly in the conditions, at the central, *F*(1.85, 31.46) = 7.94, *p* = .002, $\eta_{p}^{2}$ = .32, and left occipital, *F*(1.67, 28.41) = 5.49, *p* = .13, $\eta_{p}^{2}$ = .24, regional mean.

For each of the significant regional means, we calculated the contrasts of interest, that is, PP1 versus PP2 representing PR effects, and PP1 versus WP2 representing SR effects, for significant differences, via *t-*tests. Although, strictly speaking, the effect at the left temporal regional mean was not significant, we computed *t-*tests for this area (1) for explorative reasons, (2) because in source analysis, this regional mean might well play a role in calculating cortical sources for the activation, as well. First, the PP1 versus PP2 comparison reached significance at left occipital, *t*(17) = 2.47, *p* = .024, *d* = 0.58, and at left temporal electrodes, *t*(17) = − 3.78, *p* = .001, *d* = − 0.89. At the left occipital regional mean, the SSVEP response in the PP2 condition (*M* = 2.14 μV, SD = 1.54 μV) was decreased compared to the PP1 condition (*M* = 2.75 μV, SD = 2.11 μV). At left temporal electrodes, the SSVEP response in the PP2 condition (*M* = 0.95 μV, SD = 0.56 μV) was increased compared to the PP1 condition (*M* = 0.42 μV, SD = 0.68 μV).

Second, the PP1 versus WP2 comparison reached significance at the left occipital regional mean, *t*(17) = 2.97, *p* = .009, *d* = 0.70, and additionally at central electrodes, *t*(17) = 2.67, *p* = .016, *d* = 0.63. At both regional means the SSVEP amplitude WP2 condition (left occipital regional mean: *M* = 1.66 μV, SD = 2.43 μV; central regional mean: *M* = 0.58 μV, SD = 0.87 μV) was decreased compared to the PP1 condition (left occipital regional mean: *M* = 2.75 μV, SD = 2.11 μV; central regional mean: *M* = 0.98 μV, SD_PP1_ = 0.72 μV).

### SSVEPs in source space

**Fig. 5:**
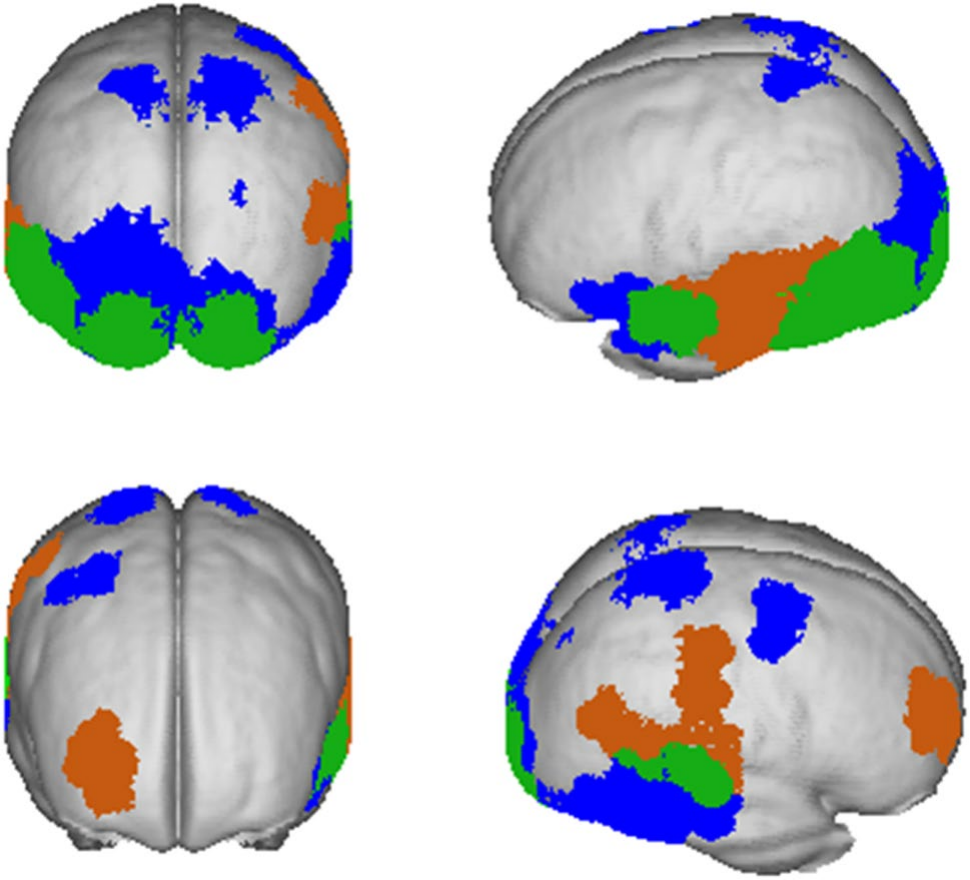
Cortical sources of the difference activity averaged in the time window from 500–2000 ms. Areas with significant activation differences below the threshold of *p* < .05 are displayed. Orange represents sources for PR (PP1-PP2), blue represents sources for SR (PP1–WP2) effects, and green represent areas with significant differences in both

Figure 5 depicts the areas that show significant activation differences in (1) PR in orange, (2) SR in blue, and (3) the overlap of both conditions (= green). The activation differences exclusively related to SR were localized in bilateral postcentral areas, bilateral occipital gyrus with a descriptively larger area in the left hemisphere, right mid frontal gyrus, left superior and inferior temporal, and right inferior via mid to superior temporal areas. The centers of gravity of the sources revealing significant activation differences are specified in Table 2.

**Table 2:** Summary of significantly activation differences and MNI coordinates of activation peaks in each region, for semantic repetition (SR), that is, the blue areas in Fig. 5

Region	Brain region	Number of grid points (total brain covers 3244)		MNI coordinates of local maxima		
				*x*	*y*	*z*
Left occipital lobe		120				
	Cuneus		26	− 50	− 69	− 17
	Lingual gyrus		25			
	Middle occipital gyrus		21			
	Inferior occipital gyrus		9			
	Fusiform gyrus		5			
	Posterior cingulate		5			
	Undefined		17			
	Areas with < 5 voxels each		12			
Right occipital lobe		39				
	Cuneus		11			
	Lingual gyrus		11	21	− 91	5
	Middle occipital gyrus		7			
	Undefined		3			
	Areas with < 5 voxels each		7			
Left temporal/frontal lobe		27				
	Superior temporal gyrus		11			
	Inferior frontal gyrus		9	− 50	10	− 17
	Areas with < 5 voxels each		5			
	Undefined		2			
Right temporal lobe		48				
	Middle temporal gyrus		19	57	− 26	12
	Superior temporal gyrus		9			
	Fusiform gyrus		7			
	Inferior temporal gyrus		6			
	Areas with < 5 voxels each		7			
Left parietal lobe		4				
	Postcentral gyrus		3	− 14	− 48	70
	Sub-gyral		1			
Right parietal lobe		11				
	Postcentral gyrus		8			
	Areas with < 5 voxels each		3	43	− 4	55
Right frontal lobe		7				
	Middle frontal gyrus		7	14	− 33	70

### Power analyses

Given a power of 0.80, an α error probability of 0.05, and *n* = 18, the minimum effect size of *d* = 0.70 can be detected. Given our effect sizes of *d* = 0.58 and − 0.89 for PR and of *d* = 0.70 and 0.63 for SR, one can conclude that the design had enough power to find effects of this magnitude—at least this holds for the effect at the left temporal Regional Mean for the PP1 versus PP2 comparison (*d* = − 0.89) and for the effect at the left occipital Regional Mean for the PP1 versus WP2 comparison (*d* = − 0.70). For the one-way repeated-measures ANOVAs, an estimated minimum effect size of *f* = 0.56, that is, $\eta_{p}^{2}$ = .24, can be detected. Therefore, one can conclude that, given our effect sizes of $\eta_{p}^{2}$ = .32, and $\eta_{p}^{2}$= .24 for the central, and left occipital regional means, respectively, our design had enough power to find effects of this magnitude.

## Discussion

In the present experiment, we recorded SSVEPs to frequency-tagged word and picture stimuli in a perceptual/semantic stimulus repetition experiment. The first aim of the study was to show that SSVEP amplitudes do not only index the processing of perceptual aspects of incoming sensory information but also semantic aspects of cortical object representations. The second aim was to specify cortical areas that are associated with the processing of semantic features of objects using the SSVEP. In a companion behavioral pre-study, we showed that both perceptual repetition (PR) and semantic repetition (SR) of flickered stimuli are associated with priming effects, indexed by faster reaction times to probe pictures no matter if primed by the same picture (perceptual priming) or the corresponding word (semantic priming). Thus, it seems safe to conclude that the present SSVEP results can be interpreted in the light of research dealing with “neuronal priming”, that is, neuronal correlates of implicit mnemonic processing.

In particular, we focused on repetition suppression as an index of sharpening processes within the neuronal networks related to perceptual/semantic priming. We found repetition suppression in PR at left occipital electrode sites. These results are in line with findings from an SSVEP study by Martens and Gruber (2012), which examined repetition-related effects during the second presentation of familiar or unfamiliar line drawings, respectively. As in our study, they observed an SSVEP amplitude suppression when repeating familiar objects (similar to PR in our study).

Furthermore, we found repetition suppression in SR. Based on these findings, we conclude that SSVEP amplitude modulations do not only reflect low-level stimulus processing within early sensory areas but also the activation of more widespread cortical networks involved in object recognition. To come to this conclusion, we argue as follows: To observe a repetition suppression effect, specific aspects of a stimulus have to be implicitly remembered during its second presentation. As we observed repetition suppression of the SSVEP during SR, that is, the trials in which a word was used to prime its associated picture, one has to conclude that this neuronal correlate of priming cannot be explained by the repetition of perceptual features. The implicitly remembered stimulus characteristic is its semantic content.

In SR, we localized the sources of the repetition effect to bilateral occipital lobes (cuneus, lingual gyrus, middle occipital gyrus), mainly the right temporal lobe (inferior, middle, and superior temporal gyrus and fusiform gyrus) and smaller parts of the left temporal lobe (superior temporal gyrus), bilateral parietal lobes (postcentral gyrus) and the left frontal gyrus. The involvement of frontal regions is in line with a model by Bar et al. which proposes that the prefrontal cortex is also involved in object processing as a “shortcut” and used for top-down processing (Bar 2003). Furthermore, SR seems to be associated with (1) retrieval of knowledge about familiar objects and (2) object processing, as we found activity in the (1) right middle frontal gyrus (Gerlach et al. 1999; Habib et al. 2003; Henson et al. 1999; Kosslyn et al. 1995) and (2) in the ventral and dorsal areas (Goodale and Milner 1992) that were associated with the respective processes in previous studies. Specifically, object recognition seems to play a role, as indicated by modulations of LOC activity (lateral occipital complex), which is considered a core region for this process (DiCarlo et al. 2012; Grill-Spector et al. 2001). Furthermore, the bilateral activation of the temporal gyrus/anterior temporal lobe probably reflects processing of semantic aspects of object representations (Bonner and Price 2013; Patterson et al. 2007). Our SR-related effects within the left inferior frontal gyrus are in line with other studies concluding that the left lateral prefrontal cortex may specifically serve as a “semantic working memory system” (for a review see Martin and Chao 2001). Furthermore, we obtained SR-related effects localized to the left anterior temporal lobe. Brambati et al. (2010) reported a similar pattern of results during the processing of unique semantic attributes (e.g., seeing the face of the president) rather than general semantic information (e.g., seeing the face of a politician). We can only speculate if the lateralized activation pattern in our study reflects the uniqueness of our stimulus material. Nonetheless, we conclude that the (LOC-) activation patterns as indexed by SSVEPs do not only mirror perceptual features, but also the processing semantic or conceptual aspects of cortical object representations.

Our results are in line with previous studies, showing that the modulation of the SSVEP signal is not limited to early sensory processes. The fact that SSVEPs reflect the activity of neuronal networks responsible for higher-level processing was, for example, shown in SSVEP studies targeting feature-based attention (Andersen et al. 2008), selective spatial attention (Andersen et al. 2011; Gundlach et al. 2020; Müller et al. 2003) and mnemonic processing (Martens and Gruber 2012). Our study complements these findings by providing evidence that SSVEPs also reflect (semantic working) memory processes. Furthermore, the present source estimates hint towards the cortical generators that are involved in the above-mentioned top-down influences during these processes.

Noteworthy, an EEG study by Friese et al. (2012) applied a very similar design as compared to our study but investigated a complementary index of neuronal processing, namely oscillatory activity in the gamma-band frequency range. They identified left temporal regions to be associated with repetition suppression effects related to semantic aspects of object representations. The fact that we identified semantic-priming-associated areas that go beyond the left temporal regions further indicates that externally triggered cortical oscillations (i.e., SSVEPs) are a useful tool to investigate complementary features of cortical object representations as opposed to internally generated oscillations (i.e., gamma-band responses).

Surprisingly, during PR, we found not only repetition suppression but also repetition enhancement. An enhancement of cortical activity is usually associated with the explicit retrieval of information (Voss and Paller 2008). This holds also for SSVEP studies showing an SSVEP increase during explicit mnemonic processes (Martens, Wahl et al. 2012a, b). Thus, we cannot exclude that our implicit memory design unintentionally induced explicit retrieval processes (explicit contamination). Regarding the general idea and interpretation of our study, this contamination poses no major problem. Nonetheless, it seems interesting that enhancement was only observed during PR and not during SR. One explanation might be provided by a study by Martens et al. (2012): In a rapid perceptual learning design, the authors presented pictures of objects twice. During the second presentation, the object could be recognized or not. Only recognized stimuli revealed SSVEP indices of implicit and explicit contributions to object recognition. Unrecognized objects (i.e., objects perceived for the first time—comparable to our probes in the SR condition) were only accompanied by SSVEP markers of implicit processes.

Notably, the observed behavioral effects do not translate 1:1 to SSVEP amplitudes. The reason for this might be twofold. First, a different group of participants took part in both experiments and the magnitude of the priming effects might differ inter-individually. Second, we cannot exclude that the perceptual priming does not include semantic processes. Thus, this condition fosters perceptual and semantic priming, plausibly resulting in stronger behavioral effects. However, the trials in which pictures were preceded by the corresponding words do not contain a repetition of perceptual features. Only the semantic aspects of the input is repeated (i.e., semantic priming).

In the present study, the stimuli were always presented at 15 Hz. However, previous studies revealed evidence that the driving frequency might have an impact on the pattern of the observed effect. For example, Kaspar et al. (2010) presented pictures of familiar and unfamiliar objects at 7.5, 12, or 15 Hz. In line with the idea that SSVEPs mirror the activation of cortical object representations, they reported higher SSVEP amplitudes elicited by familiar as opposed to unfamiliar pictures at 12 and 15 Hz. However, at a driving frequency of 7.5 Hz the effect was found to be reversed, likely to be caused by an overlap with internally generated theta rhythms. Although we used a 15-Hz pacemaker and Martens and Gruber (2012) reported a similar suppression effect at 12 Hz, we cannot conclude that the present results are robust across all driving frequencies. In future studies, it seems inevitable to examine the impact of different flicker frequencies in a parametric fashion.

In summary, our study provides further evidence that SSVEPs reflect the activation of widespread cortical networks associated with the activation of cortical object representations rather than a simple overlap of early sensory processes. This finding provides a solid basis for future studies on object recognition and mnemonic processing, which could exploit the full potential of the SSVEP technique, namely to present the constituting elements of a visual input at different driving frequencies thereby allowing for a separate analysis of the cortical processing of each element of the input (for a seminal example using the multiple-frequency tagging approach see Müller et al. 2003).

## Data Availability

Data are available upon reasonable request.
